# COVID-19 Pandemic Is a Call to Search for Alternative Protein Sources as Food and Feed: A Review of Possibilities

**DOI:** 10.3390/nu13010150

**Published:** 2021-01-05

**Authors:** Piotr Rzymski, Magdalena Kulus, Maurycy Jankowski, Claudia Dompe, Rut Bryl, James N. Petitte, Bartosz Kempisty, Paul Mozdziak

**Affiliations:** 1 Department of Environmental Medicine, Poznan University of Medical Sciences, 60-806 Poznań, Poland; 2 Integrated Science Association (ISA), Universal Scientific Education and Research Network (USERN), 60-806 Poznań, Poland; 3 Department of Veterinary Surgery, Institute of Veterinary Medicine, Nicolaus Copernicus University in Torun, 7 Gagarina St., 87-100 Torun, Poland; magdalena.kulus@umk.pl; 4 Department of Anatomy, Poznan University of Medical Sciences, 60-781 Poznań, Poland; mjankowski@ump.edu.pl (M.J.); rutbryl@gmail.com (R.B.); 5 The School of Medicine, Medical Sciences and Nutrition, University of Aberdeen, Aberdeen AB25 2ZD, UK; claudia.dompe.16@abdn.ac.uk; 6 Prestage Department of Poultry Science, College of Agriculture and Life Sciences, North Carolina State University, Raleigh, NC 27695, USA; jnppo@ncsu.edu; 7 Department of Histology and Embryology, Poznan University of Medical Sciences, 60-781 Poznań, Poland

**Keywords:** zoonosis, plant-based diet, insect-based food, cultured meat, COVID-19, SARS-CoV-2, food security

## Abstract

The coronavirus disease 2019 (COVID-19) pandemic is a global health challenge with substantial adverse effects on the world economy. It is beyond any doubt that it is, again, a call-to-action to minimize the risk of future zoonoses caused by emerging human pathogens. The primary response to contain zoonotic diseases is to call for more strict regulations on wildlife trade and hunting. This is because the origins of coronaviruses such as severe acute respiratory syndrome coronavirus 2 (SARS-CoV-2), SARS-CoV, Middle East respiratory syndrome coronavirus (MERS-CoV), as well as other viral pathogens (e.g., Ebola, HIV) are traceable to wild animals. Although COVID-19 is not related to livestock animals, the pandemic increased general attention given to zoonotic viral infections—the risk of which can also be associated with livestock. Therefore, this paper discusses the potential transformation of industrial livestock farming and the production of animal products, particularly meat, to decrease the risks for transmission of novel human pathogens. Plant-based diets have a number of advantages, but it is unrealistic to consider them as the only solution offered to the problem. Therefore, a search for alternative protein sources in insect-based foods and cultured meat, important technologies enabling safer meat production. Although both of these strategies offer a number of potential advantages, they are also subject to the number of challenges that are discussed in this paper. Importantly, insect-based foods and cultured meat can provide additional benefits in the context of ecological footprint, an aspect important in light of predicted climate changes. Furthermore, cultured meat can be regarded as ethically superior and supports better food security. There is a need to further support the implementation and expansion of all three approaches discussed in this paper, plant-based diets, insect-based foods, and cultured meat, to decrease the epidemiological risks and ensure a sustainable future. Furthermore, cultured meat also offers a number of additional benefits in the context of environmental impact, ethical issues, and food security.

## 1. Introduction

It is estimated that nearly 75% of the novel human pathogens in the last decades, the majority of which are represented by viruses, have originated in animals [1,2,3]. The pandemic of the coronavirus disease 2019 (COVID-19) has sparked an immediate discussion regarding wildlife trade and evoked various calls to ban the activity or limit it more strictly [4]. This is due to the origin of the causative factor of COVID-19, severe acute respiratory syndrome coronavirus 2 (SARS-CoV-2), which is most likely to be linked primarily with a bat host, with one study demonstrating 96% identity at the whole-genome level to betacoronavirus BatCoV RaTG13 detected in *Rhinolophus affinis* [5]. However, it is plausible that transmission to humans involved an intermediate host, and some studies have postulated that this strain evolved in pangolins [6]. Nevertheless, wild mammalian and avian species, which are often subject to hunting, can harbor strains belonging to different coronavirus genera and vary in the risk of cross-species transmission [7]. Moreover, a number of other viral pathogens posing a relevant public health threat have emerged in wild animals [4]. For example, the first case of Ebola virus infection in West Africa was likely acquired via exposure to fruit bats [8], the origin of rubella virus is probably also zoonotic (with cyclops leaf-nosed bats indicated as a primary host) [9], whereas human immunodeficiency virus 1 (HIV-1) and HIV-2 are linked to the primary transmission and further mutation of simian immunodeficiency virus from chimpanzee and sooty mangabey monkeys, respectively, during preparation and consumption of their meat [10,11].

It should be emphasized, however, that more strict regulation of hunting and wildlife trade cannot be regarded as the only strategy to prevent future zoonotic spread because the major driver of the emergence of the infectious disease includes the expansion of human settlements in more remote areas, deforestation and expansion of agricultural land, and industrialized livestock production [2,12,13]. The effect of the former substantially increased over the previous decades and will continue to grow due to the forecasted upward trend for meat. Ultimately, increased animal agriculture will contribute to an increased risk of future zoonoses.

The recent report on G4 EA H1N1 in Chinese pigs is highlighting that these animals can serve as intermediate hosts for the generation of influenza viruses with epidemic potential [14]. Furthermore, there are six coronaviruses currently known to be pathogenic to pigs. These include four members of *Alphacoronavirus* genus—porcine respiratory coronavirus (PRCoV), porcine epidemic diarrhea virus (PEDV), swine acute diarrhea syndrome-coronavirus (SADS-CoV) and transmissible gastroenteritis virus (TGEV), one member of *Betacoronavirus*—porcine hemagglutinating encephalomyelitis virus (PHEV), and one member of *Deltacoronavirus*—porcine deltacoronavirus (PDCoV) [15]. The latter has undergone the recent bird-swine transmission highlighting the potential of some deltacoronaviruses to cross species, also on the avian–mammalian routes. This is due to the fact that within avian deltacoronaviruses the recombination events frequently concern the spike region of the receptor-binding domain [16]. PEDV and SADS-CoV are also emerging swine pathogens [17]. Importantly, the latter, first identified in 2016 [18], has been recently demonstrated to infect and replicate efficiently in several different primary human lung and intestinal cells, and was not neutralized by human sera [19]. All in all, this demonstrates that not only coronaviruses related to wild animals but also to livestock can display the potential risk for future emergence events in the human population. Some mitigation can be offered by continuous surveillance and identification of new viral strains with pandemic potential [4], although this strategy itself does not offer sufficient protection

Other problems with the modern livestock industry, such as outbreaks of African swine fever (ASF) provide an additional impetus for alternative proteins. Given the current lack of vaccines or effective pharmaceuticals (although various efforts are pursued in this regard), ASF is a threat to the swine industry and food security in Europe and Asia [20]. It is also a reminder that unpredicted epidemics in livestock can cause disturbances in global food security. The other example is avian influenza that causes loss to the international poultry industry and market shares, supply shortages, trade flow disruptions and the loss of consumer confidence [21,22]. Therefore, the search for and implementation of alternative protein sources for food is urgently needed.

It should also be stressed that the range of human infectious agents related to the production of animal products goes beyond viruses and includes bacteria (e.g., mycobacteria and rickettsiae, fecal bacteria), fungi (e.g., microsporidia), parasites (helminths, metazoan, and protozoan), and prions [23]. Moreover, industrial livestock farming is associated with a need to use a variety of veterinary drugs and accounts for more than half of all antibiotic use, discharge of which promotes antibiotic resistance in the environment [24]. Last but not least, the industrial production of animal products can have devastating effects on the environment, encompassing greenhouse emissions, deforestation (including that in the Amazon River Basin, a key component of Earth’s climate system), freshwater withdrawal, eutrophication of aquatic ecosystems, and soil acidification [25].

Given all these threats associated with meat production, as well as the generally insufficient availability of high-quality meat products, especially in developing countries, it is important to consider potential alternatives to animal-derived nutrition. The adoption of plant-based products, insect sources of proteins, as well as ‘cultivated meat’ are important considerations for the future global food supply. The COVID-19 pandemic is a global public health threat but also a general reminder of severe disturbances related to zoonotic viral epidemics—the risk of which can also be associated with livestock farming. In such a broad context, it should also serve as a call-to-action to mobilize substantial resources and pursue multi-dimensional strategies to prevent future zoonosis, not only when it comes to the control of wildlife trade and consumption (the primary risk factor associated with such diseases), but also the re-evaluation of common food industry practices. The objective of this paper is to present the potential alternatives to meat production. The paper aims to answer the question of whether plant-based meats, insect-based proteins and cultured meat can provide a solution to the problem and decrease the risk of a future outbreak of zoonoses, but also to discuss the advantages and limitations to their introduction.

## 2. Is the Reduction of Meat Consumption a Solution?

Considering that meat consumption of wild or farmed origin is viewed as a potential driver for the emergence of novel zoonoses, one could suggest that its limitation may serve as a solution to minimize the risk of future spill-overs, outbreaks, and pandemics. However, achieving such a goal appears to be unrealistic. The demand for meat is facing a global increase with a worldwide surge over the last six decades [26,27]. The increasing trends are now particularly evident in developing countries. For example, India, often perceived as a household with the largest population of vegetarians [28], is currently experiencing the highest growth rates for meat consumption in the world [29]. According to the recent forecast by the World Economic Forum, global meat consumption will double by 2050. The demand for livestock products will be driven by the growth in population and in incomes, which are accompanied by changing food preferences [30]. The developed countries are still dominated by high levels of food of animal origin, and this is despite the growing appreciation of plant-based diets and the popularity of vegetarianism [31]. One should note that vegetarianism can be manifested by different forms encompassing minimizing (e.g., lacto-ovo-vegetarianism, pescetarianism) or eliminating all animal products completely from the diet (veganism), with the latter being least popular [32]. As recently highlighted, being vegan is often considered odd or deviant in the regions driven by the mainstream norms of carnism [33]. Importantly, however, limiting meat consumption has numerous positive effects beyond the epidemiological realm. They include personal health benefits, particularly in relation to limiting the consumption of red and processed meat products, which were classified as Group 2A (probably carcinogenic to humans) and Group 1 (carcinogenic to humans) by the International Agency for Research on Cancer, respectively [34]. The well-balanced plant-based diets have shown benefits in terms of prevention and treatment of cardio-metabolic disease, type 2 diabetes and decreasing the total risk of cancer [35,36,37]. On the contrary, the adherence to an unbalanced plant-based diet does come with significant health risks [38,39]. The advantage of such diets is that they are superior ethically, an aspect that plays a significant role in the decision to switch to them for some individuals [40]. Importantly, the plant-based diets are associated with a positive, decreasing effect on the ecological footprint (by lowering the demand on land, water, energy, and reducing greenhouse emissions). Such advantages cannot be ignored in light of forecasted climate changes [41]. In fact, the report on climate change and land by the Intergovernmental Panel on Climate Change (IPCC) highlights that plant-based diets are a major opportunity for mitigating and adapting to climate change—and includes a policy recommendation to reduce the consumption of meat [42].

Despite it all, plant-based products are likely not able to substitute for those of animal-origin to fulfil the goals (Table 1). This is due to the unwillingness of a relevant percentage of the human population to exclude or limit meat from their diet, challenges to develop the plant-based alternatives that mimic the livestock products in terms of organoleptic parameters and nutritional profile, but also barriers related to cost and safety (e.g., allergenicity in part of population) [43,44,45]. Furthermore, plant-based diets offer a limited alternative for meat-eating pets as they can only be applied in dogs as facultative carnivorous (although it still requires ensuring nutritional adequacy) as opposed to cats which are obligatory carnivores [46,47].

## 3. Is Insect-Based Protein a Solution?

Insects are frequently considered as pests in agriculture, disease vectors, or a nuisance for humans. However, selected species are edible whole or as an ingredient in processed food products. In fact, their consumption (known as entomophagy) has a long history and encompasses many cultures, and is still practiced in selected world regions, particularly in developing countries [48]. The edible insects are commonly consumed by an estimated one-third of the world’s population, especially in Africa, Asia and South America [49]. Although insect-based foods are currently not a part of Western diet, it is argued that their farming and use as food, and also as feed, will increase in the near future [50]. Insect-based food and feed are considered as an emerging part of the agricultural sector and as estimated, the production of insect protein in Europe can reach up to 3 million tonnes by 2030 [51,52,53]. Insects exhibit a promising nutritional profile, high protein content, considerable amount of total polyunsaturated fatty acids, reasonable levels of macro- (Ca, Na, K and P), microelements (Cu, Fe, Mn and Zn) and vitamins (A, B-group, C, D, E and K) [54]. The main insect species considered as food and feed include house cricket *Acheta domesticus*, lesser mealworm *Alphitobius diaperinus,* blue bottle fly *Calliphora vomitoria,* blow fly *Chrysomya* spp., black soldier fly *Hermetia illucens,* migratory locust *Locusta migratoria,* housefly *Musca domestica,* and tellow mealworm *Tenebrio molitor.* However, the production for direct human consumption is mostly focused on crickets and mealworms [51].

The substitution of livestock production with insect farming also has various environmental advantages such as lower greenhouse gas emission, lower withdrawal of freshwater, and less land required [55]. As noted recently by Hawkey et al., the insects mostly used for animal feed are black soldier fly larvae and mealworms [51]. The insect-based feed holds a promise to be used to feed poultry and pigs, as well as for use in aquaculture [51]. Although the use of animal-based feed in livestock farming has been prohibited in various regions due to the risks of bovine spongiform encephalitis and other transmissible spongiform encephalopathies, it should be noted that insects offer an advantage as they are considered incapable of expressing prions [51]. The ecological footprint of livestock farming would also be lowered even if insect-based products would only be applied as a feed [56].

Due to the evolutionary distance from mammals, including humans, some authors argue that insects do not pose a high zoonotic risk, contrary to livestock farming or the wild meat trade. Although insects do not have any relevant contribution to SARS-CoV-2 transmission, and there is no evidence of such a phenomenon in relation to other coronaviruses [57], recent analysis has clearly shown that these insects are a reservoir of a high diversity of RNA viruses, the majority of which are yet to be explored in terms of the potential risk to human health [58]. Additionally, the production facilities with high insect densities create a risk of rapid transmission of bacteria (e.g., Enterobacteriaceae) and parasites (e.g., *Dicrocoelium dendriticum, Gongylonema pulchrum*), which are also of concern for human health [59,60,61]. These risks can be, to some extent, mitigated via food processing (e.g., heat treatment) [62]. However, there is still a risk for workers at insect farming facilities.

Although insect-based foods can still offer some alternative to livestock meat, their market expansion is subject to several obstacles (Table 1). Firstly, as long as edible insects are considered as delicacy and part of culinary tradition in rural areas of tropical countries, the populations of the Western World associate them with plagues and health risks. This leads to a lower consumer acceptance that is challenging to overcome despite the benefits associated with the nutritional profile. On the other hand, pets readily consume insect-based foods, while insect-based pet foods could lower the ecological footprint of companion animals and decrease, partially, the pressure on livestock farming [63]. The second obstacle is due to the selective allergy of insect proteins observed in relation to edible species [51]. Another issue concerns chemical hazards originating from the contamination of substrates used for cultivation, further accumulation in insects, and deterioration of the safety of the final product. The potential number of chemicals of concern include toxic metals and metalloids, organohalogen compounds, pesticide residues, and active pharmaceutical ingredients [64,65]. The latter may also originate from the need to prevent and control the transmission of insect pathogens in large scale production facilities [66]. The potential presence of toxins produced by selected edible insect species and the accumulation of mycotoxins during the production process presents another challenge [65]. Some authors also point out the risks related to the potential harboring of antimicrobial-resistance microorganisms in edible insects [55]. Last but not least, the insect farming raises questions over specific ecological security concerns that cannot be neglected. While the sporadic escape of these organisms can be prevented, natural disasters (e.g., hurricanes, earthquakes) create a risk for a massive release, subsequent losses in agriculture, and some insect species becoming locally invasive and generating associated costs [67]. Furthermore, there is a range of insect-borne pathogens, such as picorna-like viruses, the risk of which would significantly increase with the development of intensive insect culture. This could potentially become a significant threat, particularly for the already dwindling populations of bees, the decimation of which would result in a significantly bigger ecological and agricultural disaster [68].

Although insect-based food holds promise as an alternative for livestock production, provides various benefits related to its expansion, including public health, there are still significant barriers for this industry. One should also note that the introduction of edible insects as food must be supported by the legislation and such products must be subject to the regulatory requirements, including safety assessment, that applies to all novel foods [55].

## 4. Is Cultured Meat a Solution?

Cultivated (cultured) meat is one of the terms (the others include, e.g., clean, slaughter-free meat, in vitro, lab-grown and synthetic meat, or cellular agriculture) coined to characterize the emerging technology which applies the laboratory methods of in vitro cell culture and tissue engineering to produce animal muscle under a controlled environment. Its key starting point is a collection of stem cells through animal biopsy, their further proliferation, and differentiation. The primary cells of interest include myosatellites, embryonic stem cells, adipose-derived stem cells, fibroblasts, and endothelial stem cells. Another approach is to obtain the reversibly immortalized cell lines by inducible gene expression methods (e.g., Tet-On), which can then be grown to a large scale and stored until their further differentiation is needed [69]. One should however note that methods such as Tet-On which rely on the presence of tetracycline in the culture media bring a potential risk of antibiotic residues in the final food product which consequently adds to concerns over promotion of antibiotic resistance [70]. Hence, the alternative, although ambitious, is to use induced pluripotent stem cells obtained via reprogramming of adult somatic cells [71]. One should note that this method is also not without limitations, as it relies on genetic engineering, which is still receiving low public acceptance in the food production context [72,73].

The technology of cultured meat offers a number of advantages related to public health (Table 1). In contrast to whole-animal livestock systems, it is possible to obtain the final product under controlled conditions, and prevent bacterial or fungal contamination. The risk of macroparasite presence (e.g., *Trichinella* spp., *Taenia* sp.) can be effectively mitigated. The unpredictable emergence of animal pathogens, such as ASF virus that can cause substantial economic damage, is non-existent. Cell-based manufacturing practices are predictable and less time-consuming (lasting for several weeks instead of months or years as in the case of livestock), provide a substantial benefit for ensuring food security, an aspect that cannot be ignored under the scenario of the forecasted climate crisis. Lastly, the process of cultured meat production ultimately requires sterile conditions without the use of antibiotics, which is a significant advantage given the emergence and spread of antibiotic resistance which is recognized by the World Health Organization as one of the major health threats of the 21st century [74]. This may be perceived as a challenge although one should note that in vitro cell culturing is plausible and already implemented and recommended in various laboratory studies [75,76]. The use of automated or semi-automated systems for the production of cultured meat will ensure consistent sterile conditions using high-efficiency particulate air (HEPA) filters and UV-C light [77].

The other potential benefits associated with the cultured meat is the controlled modification of the nutritional profile, e.g., by decreasing the saturated fat content or replacing it with omega-3 fatty acids [78]. However, large-scale in vitro cultures require significant effort and cost investment to be maintained, mostly concerning sourcing and preparation of adequate volumes of high-quality nutrient-rich serum, that is now commonly derived from bovine fetuses. This process poses several challenges, most notably ethical (the further need of cow farming and slaughter), as well as economical (high costs of maintaining the serums sterile and pathogen-free, as well as its supplementation with synthetic additives) [79]. The human platelet lysate, which has been demonstrated to be a viable alternative for fetal bovine serum (FBS), is economically unaffordable, particularly at the scale of cultured meat production [80,81]. One should however note that the efforts to develop and introduce non-animal substitutes to FBS, that are based on plant or mushroom ingredients, are currently being pursued [82,83,84] while some animal origin-free (AOF) media are already commercially available for cell culture, e.g., ClonaCell™-HY AOF Expansion Medium, Gibco^®^ CHO Medium or MilliporeSigma™ Chemicon™ AOF ITS.

The implementation of cultured meat is also postulated to offer a number of environmental advantages, including a significant reduction in land use and water withdrawal. It can also lead to a substantial decrease in energy consumption and greenhouse emissions under the plausible scenario of decarbonization of electricity generation [85,86]. These benefits are also present if such technology would be applied to produce the food for companion animals since meat consumption by dogs and cats also contribute to the environmental impacts [87]. However, it should be stressed that an accurate estimation of the reduction of the ecological footprint from cultured meat is currently not entirely possible as these products have yet to reach the market.

Furthermore, it also should be noted that the current methods of in vitro cultures of nutritional cells are far from optimized. While the present approaches focus on achieving a constant and continuous propagation of determined cell lines or primary cultures, this goal is still far from achieved. Hence, the current methods are characterized by relatively low yields, the need for highly specialized equipment, as well as significant energy cost. The latter is particularly important when evaluating the environmental impacts of the potential industry. While the world slowly moves towards a future based on renewable energy sources, most of the power generated nowadays comes from fossil fuels [88]. It is therefore critical to ensure that the production of cultured meat will rely on renewable energy sources—the issue that will likely also increase the public acceptance of such products but may also increase the costs of the final product. It would also be beneficial (environmentally and economically) if water employed in a process would be recaptured and reused.

Although the technology requires animals as an initial source of cells, it does not involve wholesale animal harvesting and can therefore be perceived as superior, an aspect likely having an immense contribution to consumer acceptance. Finally, it can also be used to obtain meat of rare, endangered, or locally unavailable species, as well as those which require specific qualification in meat preparation to avoid health risks (e.g., fish species belonging to Tetraodontidae family that contain lethal tetrodotoxin in internal organs and skin). Nevertheless, it should be noted that the culinary appeal of many common and rare meat types is dependent on a range of factors such as texture, accompanying elements (mostly connective and adipose, as well as the extracellular matrix), as well as the form of specific cuts. While the development of cell co-culture methods as well as novel tissue scaffolds might resolve this problem in the future, it still poses a real challenge when it comes to convincing the general public to replace certain meats with lab-grown alternatives.

In summary, coupled with the advantages discussed above and high hopes given to cultured meat as a real technological alternative to conventional meat productions, there are a number of obstacles and challenges to overcome prior to successful introduction to the market. Table 2 summarizes the challenges of cultured meat and potential mitigation strategies. One should also note that cultured meat falls into the category of novel food. Therefore, it would require a pre-market authorization, which would cover an assessment of safety profile, e.g., by the European Food Safety Authority in the European Union [89] and other regulatory agencies in other parts of the world. Such an assessment will likely cover toxicological risk and evaluation of nutritional equivalence.

## 5. Conclusions

The prevention of future zoonosis requires a multi-dimensional approach. Although the origins of SARS-CoV-2, the causative factor of COVID-19, has been linked to wild animals, the COVID-19 pandemic is a lesson on a scale of global disturbances that can be induced by zoonoses—the risk of which also exists in relation to livestock farming. While the only direct way of mitigation of risks associated with wild trade can be based on effective regulations, the underlying lack of commonly available animal-based food sources will always pose the risk of further outbreaks. Hence, while the long-term approach could focus on expanding the production of safe and controlled meat products, it would not eliminate the threats associated with livestock farming. Given the observable and forecasted global trends in meat consumption, realistic alternatives are urgently needed. Although the promotion of plant-based diets and insect-based proteins has its benefits, the highest hopes are likely to be realized with the introduction of cultured meat products. However, their success requires overcoming specific technological obstacles and social and political issues related to product acceptance. Furthermore, the underlying technology still needs to be investigated and supported economically to ensure that its potential introduction will result in lowering the environmental impact of industrial farming, especially when it comes to energy consumption. Despite that, there is no better moment than COVID-19, which is a reminder about the threats of zoonotic diseases, to mobilize resources to pursue research and development in cultured meat, gain public awareness for these products, and initiate panels on regulatory aspects of their introduction to the market.

## Figures and Tables

**Table 1:** Main advantages and disadvantages of approaches discussed in this paper as compared to conventional meat production.

Approach	Main Advantages	Main Disadvantages
Plant-based diets	Lower epidemiological risks Individual health benefits Lower ecological footprint Ethically superior	Low willingness to change the diet Allergenicity in part of the population
Plant-based substitutes		Difficulties in mimicking organoleptic properties of meat
Insect-based food	High nutritional profile Lower ecological footprint Ethically more acceptable	Risk of pathogen spread in production facilities Low consumer acceptance in various parts of the world Chemical hazards Potential escapes of insects during natural disasters The risk of viral transmissions yet to be assessed
Cultured meat	Lower epidemiological risks Predictable production Better food security Potentially lower ecological footprint Ethically superior	Number of technological obstacles The economic cost of production Public acceptance challenging to predict

**Table 2:** The main challenges for the development and commercialization of cultured meat products and potential mitigation strategies.

Challenge	Mitigation
Cell medium of non-animal origin	Use of efficient alternatives derived from plants or mushrooms
Maintaining sterility of culture without antibiotics	Good laboratory practices, aseptic techniques, a sterile work area, sterile reagents and media, good personal hygiene, sterile handling
Mimicking the texture	Use of non-animal scaffold based on polymers, safe for human consumption, e.g., alginate, chitosan, soy proteins. Incorporation of 3D-printing technology
Controlling the micronutrients profile	Addition of essential micronutrients (e.g., cyanocobalamine) to medium and ensuring their efficient cellular uptake. Use of genetically modified cell lines expressing novel biochemical pathways of nutrients synthesis
Matching the color	Addition of natural dyes (e.g., beetroot extract), extracellular hemoglobin or myoglobin, or induction of myoglobin expression under temporary lower oxygen levels
Cost-efficiency	Scaling the production to the industrial level and rapid expansion of distribution channels
Scaling the industrial production	Development of large bioreactors and associated infrastructure for cell cultures
Non-discriminatory regulations	Co-operation with regulatory bodies and associations of conventional meat producers
Consumer acceptance	Social campaigns, raising awareness on ethical, environmental, and epidemiological aspects of cultured meat.
Lowering the carbon footprint of production	Investing in dedicated, energy independent production hubs powered by renewables

## Data Availability

Not applicable.
